# A Wasteful and Harmful Practice: Sliding Scale Insulin Use Among Nursing Home Veterans

**DOI:** 10.1093/geroni/igaa057.665

**Published:** 2020-12-16

**Authors:** Kenneth Lam, Siqi Gan, Bocheng Jing, Brian Nguyen, Sei Lee

**Affiliations:** 1 VA Medical Center--San Francisco, San Francisco, California, United States; 2 University of California, San Francisco, San Francisco, California, United States; 3 San Francisco VA Medical Center, San Francisco, California, United States

## Abstract

The American Medical Directors Association and the American Diabetes Association discourage the use of sliding scale insulin (SSI) in nursing home residents with diabetes due to its association with hypoglycemia, hyperglycemia, nursing burden, and patient discomfort. However, prevalence of SSI use is unclear. We used Veterans Affairs (VA) data from October 2013 to September 2016 to determine the weekly prevalence of SSI among 22,847 veterans with diabetes admitted to VA nursing homes (NHs). Average age was 75.3 (SD 8.3) years, mean A1c was 7.3% (SD 1.6%) and 57% were admitted from hospital. We first identified residents receiving any short-acting insulin. We then classified short-acting insulin use into three mutually exclusive regimens: (1) fixed scheduled doses, (2) SSI, defined as a variable dose of short-acting insulin without a concurrent fixed dose or (3) bolus with correction (BWC), defined as a variable dose given concurrently with a fixed dose that day. During the first week of NH admission, 64.7% of residents with diabetes received no short-acting insulin, 7.4% received fixed scheduled doses, 6.3% received BWC and 21.4% were on SSI. At week 12, the prevalence of fixed dose and BWC regimens was unchanged from baseline (fixed dose = 8.4%; BWC = 7.0%). In contrast, the prevalence of SSI decreased weekly to 15.8% (p for linear trend < 0.0001). Although SSI prevalence decreased from week 1 to week 12, 51% of residents on short-acting insulin were still using SSI in their 12th week of their NH stay.

